# Multi‐UniFocality (MUF), in contrast to multifocality, in thyroid lesions: Relation to lymphocytic thyroiditis

**DOI:** 10.1111/pin.13421

**Published:** 2024-04-01

**Authors:** Mehtap Derya Aydemirli, Hans Morreau

**Affiliations:** ^1^ Department of Pathology Leiden University Medical Center Leiden the Netherlands

**Keywords:** chronic lymphocytic thyroiditis, humans, independent clonality, molecular diagnostics, multifocality, Multi‐UniFocality, next‐generation sequencing, thyroid cancer, thyroid neoplasms, thyroid nodules

## Abstract

Whereas multifocality typically concerns papillary thyroid carcinoma (PTC) without specification of intrathyroidal metastatic or independent nature of tumor foci, the designation of the latter as Multi‐UniFocal (MUF) may be relevant for select cases. A case series involving multifocal thyroid lesions with divergent histopathological morphology and/or molecular profile, with molecular evaluation of multiple individual tumor foci per patient based on a next‐generation sequencing approach, was retrospectively reviewed. Twenty‐five patient cases with multifocal thyroid lesions suggestive of MUF, with 2–6 (median 3) tumor foci per patient, were described. Tumor lesions comprised diverse histopathology, including PTC, (E)FVPTC, NIFTP, FA, FTC, and oncocytic. Morphologically similar and/or diverse tumor foci harbored different molecular alterations (suggestive of non‐shared clonality); with(out) coexistent similar foci harboring identical molecular alterations; or (partly) shared molecular alterations. MUF was associated with chronic lymphocytic thyroiditis in almost half of the cases. The recognition of MUF may justify the independent clinical consideration per individual tumor focus; as separate lesions albeit within a multifocal context. The potential clinical relevance and prognostic value of MUF remain to be further established.

**Abbreviations**
CLT: chronic lymphocytic thyroiditis
EFVPTC: encapsulated follicular variant of papillary thyroid carcinoma
FA: follicular adenoma
FTC: follicular thyroid carcinoma
FVPTC: follicular variant of papillary thyroid carcinoma
LNM: lymph node metastasis
maFA: macrofollicular adenoma
micro‐PTC: papillary thyroid microcarcinoma
miFA: microfollicular adenoma
miFTC: minimally invasive follicular thyroid carcinoma
MUF: Multi‐UniFocality
NIFTP: non‐invasive follicular thyroid neoplasm with papillary‐like nuclear features
NGS: next‐generation sequencing
PTC: papillary thyroid carcinoma
TTD: total tumor diameter

## INTRODUCTION

Unifocality and multifocality are self‐descriptive terms for the occurrence of a solitary tumor focus and multiple non‐contiguous tumor foci in the thyroid gland, respectively. Multifocality has a prevalence of 20%–40% in papillary thyroid carcinoma (PTC).^1^ However, as “multifocality” is not specified, it may be the manifestation of multicentrically occurring unifocal foci, but also of intra‐thyroidal spread, or a combination of both.^2^ This may be reflected in the clinical and prognostic importance of multifocality still being controversial.^3^ Many meta‐analytic studies have found that multifocal PTC is associated with progression and recurrence.^4^, ^5^, ^6^, ^7^ Feng et al. reported on multifocal PTC (*n* = 157, 24.7%) in their retrospective study of data from 635 PTC patients; also showing multifocality to be associated with an increased risk of recurrence, lymph node metastases, vascular invasion, and extra‐thyroidal extension, that, moreover, increased the more tumor foci were present.^8^ On the other hand, Harach et al. reported the common occurrence of occult PTC (52 foci in 36 thyroids) in their autopsy study (*n* = 101), 10 of which showed multifocality with 2–5 foci.^9^ Also, Wang et al. found in their multicenter study with over 2500 PTC patients that multifocality carried no independent risk factor.^10^ Furthermore, Harries et al., showed in their study, that multifocal and unifocal PTC had comparable rates of contralateral lobe disease, regional recurrence, and overall survival.^11^ And regarding multifocal papillary thyroid microcarcinoma (micro‐PTC), a total tumor diameter (TTD) exceeding 1 cm was associated with more aggressiveness (extra‐thyroidal extension, central/lateral lymph node metastasis) as compared with TTD ≤1 cm or unifocal micro‐PTC; with no significant difference between multifocal micro‐PTC with TTD >1 cm and multifocal/unifocal macro‐PTC.^12^

Several recent studies have focused on the further specification within multifocality. Lin et al. demonstrated the importance of distinguishing independent primary lesions from intrathyroidal metastasis in their study of 18 multifocal PTC cases: common clonality in multifocal thyroid tumors was associated with intraglandular metastatic spread, and consequently, a higher risk of lymph node metastasis and distant metastasis.^13^ In another study of eight multifocal PTC patient cases by Lu et al., nodal metastasis seemed less frequent in case of independent clonality.^1^ Also, Bansal et al. characterized multifocal PTC in 60 patient cases, reporting that multiple synchronous independent primary tumors typically occurred in different lobes, showed distinct growth patterns, and no microscopic peritumoral dissemination.^14^ And in their case report, Marín et al. described a patient with bilateral follicular variant PTC harboring a different *HRAS* and *NRAS* variant per focus, consistent with independent clonality.^15^

The recognition of unifocality within multifocal thyroid lesions (Multi‐UniFocality, MUF) may justify the independent clinical consideration per individual tumor focus; as a separate lesion albeit within a multifocal context. Accordingly, the recognition of MUF may potentially improve diagnosis and alter further clinical management, regarding (completing) thyroidectomy and/or subsequent radioactive iodine (RAI), in select cases. Still, similar studies making distinctions within multifocality, and its clinical or prognostic value, are limited.

In the present case series, the unifocal aspect in multifocal thyroid lesions is revisited through diverse clinical examples and its potential implications for management. Various cases with divergent histopathological morphology and/or molecular profile in concurrent tumor foci, suggestive of MUF, are presented. Notably, detailed molecular characterization of multiple foci per patient based on a next‐generation sequencing (NGS) approach, is provided, enabling unique insights into tumorigenic processes in MUF thyroid lesions.

## MATERIALS AND METHODS

The anonymized pathology reports of a case series involving MUF in thyroid histology or cytology slides, including molecular characterization, from 2013 through 2020, were retrospectively reviewed.

The anonymized data were handled in compliance with the Code of Conduct for the Use of Data in Health Research according to the Federation of Dutch Medical Scientific Societies (Federa), Codes of Conduct (https://www.federa.org/codes-conduct). According to the Central Committee on Research involving Human Subjects (CCMO), this type of study does not require approval from an ethics committee in the Netherlands. The study was waived by the Medical Ethics Review Committee of the Leiden University Medical Center, Leiden (decision on August 19, 2020, registration number G20.104).

Basic patient characteristics were extracted including sex, age; characteristics of the thyroid lesions including lymphocytic thyroiditis; the number, diameters, laterality/location of tumor foci, histopathological diagnoses, molecular alterations (DNA variants and/or gene fusions) along with the analysis modality used.

Multiple thyroid tumor foci with disparities in histomorphology and/or molecular characteristics, suggestive of co‐occurring independent lesions, were considered as potentially MUF. For molecular analysis, material from histological slides or occasionally cytology was morphologically selected by an experienced pathologist subspecialized in thyroid pathology.^16^ Molecular analyses were performed as part of the routine diagnostic workup in the Molecular Diagnostics Unit of the Pathology department (ISO15189 accredited) at the Leiden University Medical Center (LUMC). Nucleic acid was purified using a fully automated DNA/RNA isolation system.^17^

Molecular diagnostics using NGS for somatic gene variant (with a custom AmpliSeq™ Cancer Hotspot Panel; Thermo Fisher Scientific) and/or gene fusion analysis (with Archer® FusionPlex® CTL panel; ArcherDX Inc.) were described previously.^18^, ^19^, ^20^ From 2013 through 2015/2016, a hotspot mutation analysis using Taqman hydrolysis assay was used.^21^ From 2015, a custom AmpliSeq Cancer Hotspot Panel (CHSP) was used with frequent updates. The CHSPv2/v3/v4/v6 targets 50/60/74/85 genes, respectively. The Archer FusionPlex CTL panel targeted 36 genes and was used from 2016 on. Molecular diagnostic results were interpreted by registered molecular scientists in pathology.

Contributing immunohistochemical analysis results of VE1, a *BRAF* ^*V600E*^ mutation‐specific antibody (clone VE1),^22^ were also retrieved from the pathology reports.

## RESULTS

The pathology reports of 25 patients with thyroid lesions involving diverse presentations of potential MUF were reviewed, see Table 1 for characteristics. The case series, including histopathology and molecular data per tumor focus, is presented in Table 2. For full mutational data, see Supporting Information: Table S1, and for a histological impression, see Supporting Information: Figure S1.

**Table 1:** Characteristics.

Parameters	Patients, *n* = 25 (100%)
Female, *n* (%)	17 (68)
Age, years (median, range)	47 (29–71)
Tumor foci per patient (median, range)	3 (2–6)
Tumor size, *n* (%)	
≤1 cm	51 (62)
>1 cm	31 (38)
Chronic lymphocytic thyroiditis, *n* (%)	12 (48)
Lymph node metastasis, *n* (%)	2 (8)
Distant metastasis, *n* (%)	1 (4)
Total thyroidectomy, *n* (%)	20 (80)^a^
Unilateral lesions, *n* (%)	12 (50)
Bilateral lesions, *n* (%)	12 (50)

Parameters	Tumor foci, *n* = 86 (100%)
Histology, *n* (%)	
PTC (including micro‐PTC)	33 (38)
micro‐PTC	26 (30)
FVPTC	14 (16)^b^
EFVPTC	4 (5)
NIFTP	13 (15)
FA	19 (22)^c^
Oncocytic proliferation focus	1 (1)
miFTC	2 (2)

*Note* 1: as regards subtyping of papillary thyroid (micro)carcinoma (PTC): if not further specified, this concerns “classic papillary thyroid carcinoma” in this manuscript.

*Note* 2: the designation of papillary thyroid microcarcinoma (micro‐PTC) was used to emphasize subcentimetric PTC foci with a diameter ≤1 cm due to potential clinical implications in the current context, rather than a histological subtype.

Abbreviations: EFVPTC, encapsulated follicular variant of papillary thyroid carcinoma; FA, follicular adenoma; FVPTC, follicular variant of papillary thyroid carcinoma; maFA, macrofollicular adenoma; micro‐PTC, papillary thyroid microcarcinoma; miFA, microfollicular adenoma; miFTC, minimally invasive follicular thyroid carcinoma; NIFTP, non‐invasive follicular thyroid neoplasm with papillary‐like nuclear features; PTC, papillary thyroid carcinoma.

^a^ Total thyroidectomy, either single‐ or two‐staged, was performed in nearly all but five patients (hemithyroidectomy in cases 13, 22, 23, 24, and 25).

^b^ Partly oncocytic in one FVPTC focus.

^c^ Including 2 macrofollicular adenoma (maFA), 1 microfollicular adenoma (miFA), and 1 oncocytic FA.

**Table 2:** Case series of patients with thyroid lesions involving Multi‐UniFocality (MUF).

ID	Sex	Age (yrs)	CLT	Tumor focus	Histopathology	Somatic molecular alteration	Diam. (mm)	Loc.
**Similar/diverse histopathology and divergent molecular alterations (MUF)**								
1	f	36	+	T1	PTC	*CCDC6‐RET*	18	R
				T2	Micro‐PTC	*BRAF* ^*V600E*^	8	R
		37	+	T3	Micro‐PTC		2.5	L
				T4	Micro‐PTC		1	L
2	f	47	+	T1^a^	Micro‐PTC	*BRAF* ^*V600E*^	1	R
				T2	Micro‐PTC	*SASH1‐BRAF*	1.5	R
				T3	Micro‐PTC	*BRAF* ^*non‐V600E*^	7	R
				T4	maFA		22	R
3^b^	m	35	+	T1	PTC	*MAP2K1*	70	R
				T2	Micro‐PTC	*BRAF* ^*V600E*^	2	L
4	f	41	+	T1	FVPTC	*PAX8‐PPARG*	57	L
				T2	FVPTC	*NRAS* ^*Q61K*^	2.3	L
		41	+	T3	FVPTC	*SCD5‐MET*	15	R
				T4	FVPTC		11	R
				T5	FVPTC		7	R
5	m	47	–	T1	FVPTC	*NRAS* ^*Q61K*^	8	L
				T2	FVPTC partly oncocytic	*NRAS* ^*Q61R*^	2	L
		47	–	T3	FVPTC	*KRAS* ^*Q61R*^	3	R
6	f	49	–	T1	FA/NIFTP	*NRAS* ^*Q61K*^	10	R
				T2	FA	*PAX8‐PPARG*	15	L
7	f	58	–	T1	Micro‐PTC	*BRAF* ^*V600E*^	6	R
				T2	NIFTP	*NRAS* ^*Q61R*^	7	R
				T3	NIFTP	*HRAS* ^*Q61K*^	10	R
		58	–	T4	NIFTP		3	L
8	f	61	–	T1	FVPTC	*NRAS* ^*Q61R*^	6	R
				T2	FVPTC	*TP53*	6	R
				T3	Micro‐PTC		2	R
				T4	Micro‐PTC		2	R
				T5	Micro‐PTC		2	R
				T6	Micro‐PTC		2	R
9	f	47	+	T1	FA		10	L
				T2	PTC			L
		51	+	T3	Micro‐PTC		9	R
				T4	miFTC	*NRAS* ^*Q61R*^	12	R
10	m	35	–	T1	EFVPTC	*NRAS* ^*Q61R*^	38	R
				T2	Micro‐PTC	*BRAF* ^*V600E*^	1.5	R
11	m	51	–	T1^a^	Micro‐PTC	*BRAF* ^*V600E*^	4	R
				T2	EFVPTC	*NRAS* ^*Q61R*^	19	R
				T3	FA	No variant detected	34	R
12^b^	m	29	–	T1	PTC	*BRAF* ^*V600E*^	15	R
				T2	NIFTP	*KRAS* ^*Q61R*^	24	L
13	m	52	–	T1	EFVPTC	*KRAS* ^*G12R*^	15	R
				T2	Micro‐PTC	No variant detected	1.1	R
14	m	66	+	T1^a^	FVPTC	*NRAS* ^*Q61R*^	40	L
				T2^a^	FA oncocytic	*HRAS* ^*Q61K*^	17	R
				T3	maFA		20	R
15	f	29	+	T1	FA	No fusion/variant detected	17	R
				T2	Micro‐PTC	*BRAF* ^*V600E*^	10	I
				T3	Micro‐PTC		3	I
				T4	Micro‐PTC		1	I
				T5	Micro‐PTC		1.5	L
**MUF and coexisting foci of similar morphology with identical molecular alterations**								
16	f	56	–	T1	NIFTP	*NCOA6‐PPARG*	30	L
				T2	Micro‐PTC	*KRAS* ^*Q61K*^	3	L
				T3	Micro‐PTC	*KRAS* ^*Q61K*^	2	L
17	f	52	+	T1	NIFTP	*NRAS* ^*Q61K*^	18	
				T2	NIFTP	*NRAS* ^*Q61K*^	16	
				T3	FA	*KRAS* ^*Q61R*^	13	
18	f	44	–	T1^a^	PTC	*BRAF* ^*V600E*^	45	R
				T2	FA	*PTEN* ^*D252V*^	6	R
				T3^a^	PTC	*BRAF* ^*V600E*^	11	R
19	m	34	+	T1	FVPTC	*ETV6‐NTRK3*	7	R
				T2	FVPTC	*ETV6‐NTRK3*	8	R
				T3	FA	No fusion detected		R
				T4	miFA	No variant detected	12	R
20	f	46	+	T1	Micro‐PTC	*BRAF* ^*V600E*^	10	R
				T2	Micro‐PTC	*BRAF* ^*V600E*^	10	R
				T3	Micro‐PTC	*BRAF* ^*V600E*^	5	R
				T4	FA	No variant detected	5	R
		47	+	T5^a^	PTC	*BRAF* ^*V600E*^	12	L
21	f	60	–	T1	EFVPTC	*HRAS* ^*Q61K*^	20	R
		71	–	T2	NIFTP	*NRAS* ^*Q61R*^	10	L
				M1	FVPTC	*HRAS* ^*Q61K*^		L3^c^
**(Partly) shared molecular alterations (progression/subclonal diversification/other?)**								
22	f	61	–	T1	FA	*KRAS* ^*Q61R*^	25	L
				T2	Oncocytic proliferation^d^	*KRAS* ^*Q61R*^ and *TERTp*	17	L
				T3	NIFTP	*NRAS* ^*Q61R*^ and *SMAD4*	2	L
23	f	38	+	T1	miFTC	*HRAS* ^*Q61R*^	10	R
				T2	FA	*HRAS* ^*Q61R*^	10	R
				T3	FA	*HRAS* ^*Q61R*^	10	R
				T4	FA	*NRAS* ^*Q61R*^	19	R
				T5	FA	*NRAS* ^*Q61R*^	10	R
				T6	FA		5	R
24	f	38	–	T1a	NIFTP high cellularity	*HRAS* ^*Q61R*^	19	R
				T1b	NIFTP low cellularity	*HRAS* ^*Q61R*^		R
25	f	55	+	T1	NIFTP high cellularity	*HRAS* ^*Q61R*^	7	R
				T2	NIFTP low cellularity	*HRAS* ^*Q61R*^	7	R
				T3	Micro‐PTC		1.6	R

*Note* 1: as regards subtyping of papillary thyroid (micro)carcinoma (PTC): if not further specified, this concerns “classic papillary thyroid carcinoma” in this manuscript.

*Note* 2: the designation of papillary thyroid microcarcinoma (micro‐PTC) was used to emphasize subcentimetric PTC foci with a diameter ≤1 cm due to potential clinical implications in the current context, rather than a histological subtype.

Abbreviations: EFVPTC, encapsulated follicular variant of papillary thyroid carcinoma; FA, follicular adenoma; FVPTC, follicular variant of papillary thyroid carcinoma; maFA, macrofollicular adenoma; micro‐PTC, papillary thyroid microcarcinoma; miFA, microfollicular adenoma; miFTC, minimally invasive follicular thyroid carcinoma; NIFTP, non‐invasive follicular thyroid neoplasm with papillary‐like nuclear features; PTC, papillary thyroid carcinoma.

+, present; –, absent; f, female; m, male; yrs, years; CLT, chronic lymphocytic thyroiditis; Diam., diameter; Loc., location; T, tumor focus; M, metastatic focus; L, left lobe of thyroid gland; I, isthmus; R, right lobe of thyroid gland.

^a^ The BRAF status was determined using immunohistochemistry in three cases (T1 of case 2; T1 of case 11; T1 (additional molecular diagnostics) and T3 of case 18). The molecular diagnostic results of three tumor foci (T1 and T2 of case 14; T5 of case 20) were obtained from the thyroid cytology slide.

^b^ Lymph node metastasis present in cases 3 and 12.

^c^ Lumbar vertebra L3.

^d^ Inside the FA (T1) there was an oncocytic proliferation (T2) (lack of thick capsule or capsular/vascular invasion, but aggressive molecular profile; hence no designation as either oncocytic follicular adenoma or oncocytic carcinoma of the thyroid, was preferred in this specific case).

In general, various cases had multiple tumor foci with similar and/or diverse histopathology with different molecular alterations in many tumor foci (cases 1–15). The morphological findings and/or molecular data with different DNA variants and/or gene fusions suggest non‐shared clonality in many lesions; thus the simultaneous occurrence of multiple independent foci. Some cases (cases 16–21) additionally had coexisting lesions of similar morphology with identical molecular alterations (suggestive of coexistent shared clonality/intrathyroidal metastasis). Some (cases 22–25) involved tumor foci with (partly) shared molecular alterations.

Almost half of the cases with MUF had coexistent chronic lymphocytic thyroiditis (CLT). For a comparison of patient characteristics based on coexistent CLT, see Supporting Information: Table S2.

Potential clinical implications may ensue, based on the assumption that MUF could potentially justify the independent consideration of the individual prognostic value of each distinct tumor focus. Of course, markers of aggressiveness/invasiveness, other clinical considerations, and current guidelines should be taken into account in the overall assessment.

As such, in some cases of MUF micro‐PTCs, for example, case 2 with three unilateral MUF micro‐PTCs, conservative (expectant) management could possibly be advocated, instead of a (completing) thyroidectomy (and subsequent RAI). The dissimilar cancer driver mutations would, in spite of corresponding histopathological morphology, suggest independent origination of the individual tumor foci. Notwithstanding combined diameters exceeding 1 cm, it could be advocated to consider the separate foci as multiple independent (or MUF) micro‐PTCs, with their corresponding (conservative) clinical management plan, rather than assessment based on TTD.

In bilateral lesions showing MUF, alterations in RAI strategies might be considered.

Our NGS analysis‐based approach for molecular evaluation per tumor focus provides a comprehensive understanding of MUF and tumorigenesis processes in thyroid tumors, for example, due to divergent and/or (partly) shared identical molecular alterations.

Case 21, for example, involved a previously resected EFVPTC lesion carrying an *HRAS* variant, where biopsy of the lumbar vertebra L3 eleven years later showed a metastatic thyroid cancer lesion morphologically similar to the prior EFVPTC and identical *HRAS* variant. The residual thyroid lobe also contained a lesion, whereupon completing hemithyroidectomy was performed, revealing an NIFTP focus with an *NRAS* variant. These findings suggest that the current L3 lesion probably was an old metachronous metastasis, whereas the present NIFTP a MUF tumor focus unconnected to the metastasis.

Case 22 involved an NIFTP focus with coexistent *NRAS and SMAD4* variants, and a FA focus with a *KRAS* variant. Remarkably, within the FA, there was a focus of oncocytic proliferation that carried the same identical *KRAS* variant and in addition a *TERTp* variant; suggestive of subclonal diversification with progression into another histopathological morphology with an aggressive molecular pattern (gain of a *TERTp* mutation^23^); next to a clonally unrelated lesion (NIFTP vs FA/oncocytic proliferation).

In case 23, there were five FA foci and one miFTC focus, all occurring in the context of CLT. Three of these foci, including the miFTC, harbored an identical *HRAS* variant. Two of the others harbored an identical *NRAS* variant. The biggest change is that the similarity of the variants has occurred pure by chance, as the morphology indicates a non‐clonal relation. One of the lesions might have progressed (FA into miFTC).

Of note, case 24 (without CLT) involved a tumor consisting of two sharply demarcated NIFTP components with morphological disparity, that is, high versus low cellularity, while both harbored identical *HRAS* gene variants, most likely indicating the presence of two morphological components of the same NIFTP lesion. In contrast, case 25 (with CLT) showed two separate NIFTPs (next to a micro‐PTC) with identical *HRAS* gene variants, however may be considered as MUF foci inherent to their nature and circumscribed aspect of the nodules.

## DISCUSSION

The present case series shows that MUF rather transcends beyond the definition of classic multifocal PTC, as it indicates the presence of independent unifocal thyroid lesions. MUF is the designation of likely non‐clonal independent tumor foci, suggested by divergent histopathology and/or molecular profile. An impression of the variety and (potential) clinical relevance is given using several MUF cases, comprising diverse histopathological morphology including PTC, but also FA, FTC, oncocytic proliferation, NIFTP, (E)FVPTC.

Tumor foci were of similar and/or diverse histopathological morphology with different molecular alterations; suggestive of non‐shared clonality. Some cases additionally had coexistent likely metastatic foci of similar morphology harboring identical molecular alterations. Other cases had foci with (partly) shared molecular alterations including subclonal diversification.

### Recognition of MUF: Providing insights into mechanisms of tumorigenesis

In particular, an NGS‐based approach for molecular evaluation per tumor focus allowed for a comprehensive understanding of MUF and tumorigenesis processes. Progression into another histopathological morphology, FA into miFTC, was suggested by shared identical molecular alterations in coexisting foci in one patient case. Or, FA into an oncocytic proliferation additionally with a more aggressive molecular pattern.

### Recognition of MUF: Potential consequences for clinical management

The recognition of MUF may allow for adapting the further management strategy in specific clinical cases, assuming MUF may potentially justify the independent consideration (of the individual prognostic value) of each distinct tumor focus. For a graphical impression, also see Supporting Information: Figure S2.

In current clinical practice, total thyroidectomy with subsequent RAI is often recommended for low‐risk and intermediate‐risk PTCs, and usually multifocality is attributed as a rationale for this treatment strategy. Current evidence indicates multifocal micro‐PTC with a TTD of >1 cm as more aggressive than multifocal/unifocal PTC ≤1 cm^12^; however, the reflection of MUF in this, as opposed to intrathyroidal metastasis, is unknown.

Besides, several studies concluded that hemithyroidectomy alone may be a safe treatment option for selected patients with multifocal PTC,^24^, ^25^, ^26^ and MUF may hypothetically be a potential underlying factor.

As such, an expectant management strategy could be advocated (diagnostic hemithyroidectomy), instead of a (completing) thyroidectomy with subsequent RAI, for MUF unilateral micro‐PTCs, regardless of whether their combined diameters exceed 1 cm. Due to separate assessments per MUF focus, rather than taking them together to the TTD, these could still be regarded as low‐risk entities with their corresponding conservative management.

Further, alterations in RAI strategies may be considered following total thyroidectomy in those cases with bilateral lesions considered low‐risk due to MUF.

In principle, the treatment strategy can be altered, due to MUF foci being considered as independent lesions. It should however be stressed that each patient case requires an individual evaluation within their respective clinical context, in the absence of adverse features (e.g., extrathyroidal extension [lymph node/distant] metastasis), while considering current guidelines.^27^

### Considerations regarding the identification of MUF: Molecular evaluation and interpretation

Care should be taken to identify MUF correctly. MUF is not fully interchangeable with non‐shared clonality, subclonal diversity, nor entirely excluded by shared clonality or shared molecular alterations.

Characteristics of molecular alterations involved require careful evaluation, such as the presence of gene variants that are associated with progression or an aggressive nature (e.g., *TERTp*, *TP53)*.^23^, ^28^ Identical mutations with highly frequent occurrence, for example, classic *BRAF* ^V600E^ mutation in PTC,^23^ in multiple tumor foci, could indicate shared clonality due to intrathyroidal metastatic spread, yet their independent unifocal origination may also be a plausible alternative explanation.^1^ Common oncogenic factors including genetic, environmental, and epidemiological, may be exposing a risk for the emergence of multiple tumors (field cancerization^29^), whereby susceptible cells are transformed simultaneously leading to parallel oncogenesis.^30^ Multiple foci sharing a rare variant would more readily suggest a shared clonal origin, rather than independent unifocal origins, though also here shared identical molecular alterations by pure chance cannot be excluded.

Other considerations, although uncommon, include the differentiation of MUF from “tumor‐to‐tumor metastasis”; which may also present with divergent morphology (metastatic deposit with its respective histopathology) in a thyroid neoplasm.^31^, ^32^

### Chronic lymphocytic thyroiditis

Among the presented patient cases with MUF, almost half had a background of CLT. In comparison: in other studies, 0.5%–38% of PTC had coexistent CLT,^33^, ^34^, ^35^, ^36^ and in the recent MASTER study on approximately thousand low to intermediate‐risk PTC patients, 10% had coexistent CLT, while multifocality was an independent factor associated with CLT.^37^ Other studies have also associated CLT with multifocality in papillary thyroid (micro)carcinomas.^34^, ^38^, ^39^, ^40^, ^41^ Indeed, CLT has been associated with a higher risk of thyroid cancer, as a carcinogenesis‐promoting condition.^39^, ^42^

At the same time, several studies have found concurrent CLT to be associated with favorable clinical outcomes (better prognosis, lower recurrence rates) and a protective effect even,^41^, ^43^, ^44^, ^45^ while other studies found no association.^35^, ^36^, ^46^ In addition, coexistent CLT was negatively correlated with angiolymphatic invasion and (central) lymph node metastasis (LNM) in few studies.^37^, ^41^ Multifocality was associated with central LNM in the patients with coexistent CLT,^43^ but again, MUF is not differentiated from the intrathyroidal metastatic lesions within multifocality. In the MASTER study on low to intermediate‐risk PTC patients, independent risk factors for central LNM included: male sex, age under 55 years, tumor diameter >1 cm, extrathyroidal extension, angiolymphatic invasion, but not multifocality.^37^ Furthermore, only angiolymphatic invasion remained an independent risk factor in the subset of patients with coexistent CLT.^37^

In our study, two young male patients with and without CTL showed LNM (patients 3 and 12, resp.) and in one male patient without CLT distant metastasis was seen (patient 21). In most clinical practices, it is not easy to perform clonality assays as presented in this manuscript. A rule of thumb may be that the chance of MUF might be far higher in cases with CLT and may influence clinical decision‐making. However, a careful assessment per individual case is essential. Although suggestive to be an important factor in MUF the association between the presence of (extensive) CLT and MUF also needs further study.

### Other studies: Alternative denominations, clonality assessments, and molecular profiling

Alternative denominations for MUF in PTC include true multicentricity, multicentric origin, multiple synchronous primary tumors (MSPTs),^14^ or independent primary (IP) PTC, as separated from intrathyroid metastatic (ITM) PTC,^13^ in multifocal PTC. However, diverse histopathology, both syn‐ and metachronous tumors (e.g., patient 21), are also covered by MUF, besides the linguistic and contextual relation to multifocality.

Several studies assessed clonality status in multifocal PTC based on X chromosome‐inactivation patterns using HUMARA,^47^, ^48^, ^49^, ^50^ combined with LOH,^51^ or BRAF analysis^52^, ^53^; the latter two^13^, ^54^; or multiple mutations/fusions.^14^ Few studies explored clonality in multifocal PTC using *BRAF* analysis only,^55^, ^56^ *RET*/PTC‐1, ‐2, ‐3 only,^57^ or additional miRNA profiling.^58^ Multiple mutations (*BRAF*, *NRAS*, *HRAS*, *KRAS*), and gene rearrangements (*RET*/PTC1, *RET*/PTC3) were tested for in multifocal PTC in the study by Bansal et al.^14^

More recently, an NGS‐based approach was applied by Marín et al. in their patient case involving two encapsulated FVPTC foci (*HRAS* and *NRAS* variant, respectively) in contralateral lobes^15^; A similar case is described in the present series. Lu et al. implemented whole exome sequencing and targeted region sequencing, providing insight on tumorigenesis with extensive analyses in eight multifocal PTC cases featuring likely common clonal foci, but also likely independent clonal foci whether or not accompanied by the former^1^; in line with findings in the present report.

As regards laterality, see “Additional discussion” in the Supporting Information.

### Limitations and other considerations as regards molecular analysis

A limitation of our study is the relatively limited number of cases and non‐structural collection of cases. Consequently, no statement can be made regarding the incidence of MUF or a potential correlation with certain factors, based on our case series by default. Similarly, as not every single tumor focus found on histopathological examination receives (full) molecular analysis, potential cases of MUF go undiscovered. Vice versa, the evaluation of detected molecular alterations in combination with histopathology may be suggestive of MUF, whereas possibly undetected molecular alterations might have changed the overall interpretation. Also, (molecular) findings may be suggestive of the likelihood of clonal relatedness, though not deduced with full certainty. Complete molecular characterization of every tumor focus is mostly not performed, feasible, or clinically relevant; although it may be informative. The costs associated with the use of NGS limit its profuse use. Surely, in case of multifocal PTC, *BRAF* ^*V600E*^ immunohistochemistry could serve as an initial means of screening, based on prevalence. However, specific clinical cases subject to certain decisions in management strategy could potentially benefit from the specification of multifocality as MUF in particular.

## CONCLUSION

Whereas classic multifocality typically concerns PTC and has no further specification in terms of intrathyroidal metastatic or independent nature of tumor foci, the designation of the latter as MUF may bring the critical nuance essential to select cases. Divergent histopathological morphology and/or molecular profile in concurrent tumor foci may be suggestive of MUF. The recognition of MUF may justify the independent clinical consideration per individual tumor focus; as a separate lesion albeit within a multifocal context. Several studies concluded that hemithyroidectomy alone may be a safe treatment option for selected patients with multifocal PTC and MUF may hypothetically be a potential underlying factor. MUF is often seen in the context of CLT. This case series further adds to the knowledge on tumorigenesis processes in MUF thyroid lesions, by providing extensive molecular information on multiple foci per patient based on an NGS approach. The potential clinical relevance and prognostic value remain to be further established.

## AUTHOR CONTRIBUTIONS

Hans Morreau: Conceptualization; methodology. Mehtap Derya Aydemirli: Visualization; data extraction/tabulation. Mehtap Derya Aydemirli and Hans Morreau drafted, edited, and reviewed the final manuscript.

## CONFLICT OF INTEREST STATEMENT

Hans Morreau: Advisor GenomeScan, Leiden, The Netherlands. The authors declare no conflict of interest that could be perceived as prejudicing the impartiality of this study.

## ETHICS STATEMENT

The anonymized data was handled in compliance with the Code of Conduct for the Use of Data in Health Research according to the Federation of Dutch Medical Scientific Societies (Federa), Codes of Conduct (https://www.federa.org/codes-conduct). According to the Central Committee on Research involving Human Subjects (CCMO), this type of study does not require approval from an ethics committee in the Netherlands. The study was waived by the Medical Ethics Review Committee of the Leiden University Medical Center, Leiden (decision on August 19, 2020, registration number G20.104).

## Supporting information

Additional Supporting Information may be found in the online version of this article at the publisher's website.

Supporting information.

Supporting information.

## Data Availability

The data that support the findings of this study are available in the table of this article.
