# Supplementary material for: Multi‐UniFocality (MUF), in contrast to multifocality, in thyroid lesions: Relation to lymphocytic thyroiditis
Source: Pathol Int. 2024 Apr 1;74(5):274–84. doi: 10.1111/pin.13421 (PMC11551814; doi:10.1111/pin.13421)
Supplement: Supplementary file 1 — Supporting information. [file PIN-74-274-s001.pdf]

Supplementary material

Multi-UniFocality (MUF), in Contrast to Multifocality, in Thyroid Lesions: Relation to Lymphocytic Thyroiditis

Aydemirli MD, Morreau H*

*Corresponding author: Prof. dr. Hans Morreau, J.Morreau@lumc.nl

Supplementary Table S1. Case series of patients with thyroid lesions involving Multi-UniFocality (MUF), full data

ID	Sex	Age (yrs)	CLT	Tumor focus	Histopathology	Full somatic mutational data	Diam. (mm)	Loc.	CHSP/ Taq/IHC	Archer CTL FusionPlex
					**Similar/diverse histopathology & divergent molecular alterations (MUF)**					
1	f	36	+	T1	PTC	*CCDC6*: NM_005436: exon:1; *RET*: NM_020630: exon:11	18	R		Archer
				T2	micro-PTC	*BRAF* (exon 15), NM_004333.4: c.1799T>A, p.(Val600Glu)	8	R		Archer
		37		T3	micro-PTC		2.5	L		
				T4	micro-PTC		1	L		
2	f	47	+	T1	micro-PTC	*BRAF*^{V600E}	1	R	IHC	
				T2	micro-PTC	*SASH1*: NM_015278.3:exon:2; *BRAF*: NM_004333.4:exon:11	1.5	R	CHSPv4	Archer
				T3	micro-PTC	*BRAF* (exon 15), NM_004333.4: c.1798_1810delinsAGTG, p.(Val600_Trp604delinsSerGly)	7	R	CHSPv4	Archer
				T4	maFA		22	R		
3‡	m	35	+	T1	PTC	*MAP2K1* (exon 3); NM_002755.3: c.303_311delinsCAG, p.(Glu102_Lys104delinsArg)	70	R	CHSPv6	Archer
				T2	micro-PTC	*BRAF* (exon 15): NM_004333.4: c.1799T>A, p.(Val600Glu)	2	L	CHSPv6	Archer
4	f	41	+	T1	FVPTC	*PAX8*(-) exon 8 , NM_003466.3 ; *PPARG*(+) exon 2, NM_005037.5	57	L	CHSPv2	Archer
				T2	FVPTC	*NRAS* (exon 3), NM_002524.4: c.181C>A, p.(Gln61Lys)	2.3	L	CHSPv2	
		41		T3	FVPTC	*SCD5*(-) exon 1, NM_024906.2 ; *MET*(+) exon 2, NM_000245.2	15	R	CHSPv3	Archer
				T4	FVPTC		11	R		
				T5	FVPTC		7	R		
5	m	47	-	T1	FVPTC	*NRAS* (exon 3), NM_002524.4: c.181C>A, p. (Gln61Lys)	8	L	Taq	
				T2	FVPTC partly oncocytic	*NRAS* (exon 3), NM_002524.4: c.182A>G, p.(Gln61Arg)	2	L	Taq	
		47		T3	FVPTC	*KRAS* (exon 3), NM_004985.3: c.182A>G, p. (Gln61Arg)	3	R	Taq	
6	f	49	-	T1	FA/NIFTP	*NRAS* (exon 3); : NM_002524.4: c.181C>A, p. (Gln61Lys)	10	R	CHSPv4	Archer
				T2	FA	*PAX8*: NM_003466.3:exon:2 ; *PPARG*: NM_005037.5:exon:2	15	L		Archer
7	f	58	-	T1	micro-PTC	*BRAF* (exon 15), NM_004333.4: c.1799T>A, p.(Val600Glu)	6	R	CHSPv3	
				T2	NIFTP	*NRAS* (exon 3), NM_002524.4: c.182A>G, p. (Gln61Arg)	7	R	CHSPv3	
				T3	NIFTP	*HRAS* (exon 3), NM_001130442.1: c.181C>A, p. (Gln61Lys)	10	R	CHSPv3	
		58		T4	NIFTP		3	L		
8	f	61	-	T1	FVPTC	*NRAS* (exon 3), NM_002524.4: c.182A>G, p.(Gln61Arg)	6	R	CHSPv2	
				T2	FVPTC	*TP53* (exon 6), NM_000546.5: c.638G>A, p.(Arg213Gln)	6	R	CHSPv2	
				T3	micro-PTC		2	R		
				T4	micro-PTC		2	R		
				T5	micro-PTC		2	R		
				T6	micro-PTC		2	R		
9	f	47	+	T1	FA		10	L		
				T2	PTC			L		
		51	+	T3	micro-PTC		9	R		
				T4	miFTC	*NRAS* (exon 3): NM_002524.4 c.182A>G, p.(Gln61Arg)	12	R	CHSPv4	
10	m	35	-	T1	EFVPTC	*NRAS* (exon 3), NM_002524.4: c.182A>G, p.(Gln61Arg)	38	R	Taq	
				T2	micro-PTC	*BRAF* (exon 15), NM_004333.4: c.1799T>A, p.(Val600Glu)	1.5	R	Taq	
11	m	51	-	T1	micro-PTC	*BRAF*^{V600E}	4	R	IHC	
				T2	EFVPTC	*NRAS* (exon 3); : NM_002524.4: c.182A>G, p.(Gln61Arg)	19	R	CHSPv6	
				T3	FA	No variant detected	34	R	CHSPv6	
12‡	m	29	-	T1	PTC	*BRAF* (exon 15), NM_004333.4: c.1799T>A, p. (Val600Glu)	15	R		Archer
				T2	NIFTP	*KRAS* (exon 3), NM_004985.3: c.182A>G, p. (Gln61Arg)	24	L		Archer
13	m	52	-	T1	EFVPTC	*KRAS* (exon 2): NM_004985.3: c.34G>C, p.(Gly12Arg)	15	R	CHSPv4	
				T2	micro-PTC	No variant detected	1.1	R	CHSPv4	
14	m	66	+	T1	FVPTC	*NRAS* (exon 3), NM_002524.4: c.182A>G, p.(Gln61Arg)	40	L	CHSPv4†	
				T2	FA oncocytic	*HRAS* (exon 3), NM_001130442.1: c.181C>A, p.(Gln61Lys)	17	R	CHSPv4†	
				T3	maFA		20	R		
15	f	29	+	T1	FA	No fusion/variant detected	17	R	CHSPv2	Archer
				T2	micro-PTC	*BRAF* (exon 15), NM_004333.4: c.1799T>A, p.(Val600Glu)	10	I	CHSPv2	
				T3	micro-PTC		3	I		
				T4	micro-PTC		1	I		
				T5	micro-PTC		1.5	L		

**MUF & coexisting foci of similar morphology with identical molecular alterations**										
16	f	56	-	T1	NIFTP	*NCOA6* (-) exon 9, NM_014071.3 ; *PPARG*(+) exon 2, NM_005037.5	30	L	CHSPv2	Archer
				T2	micro-PTC	*KRAS* (exon 3), NM_033360.2: c.180_181delTCinsAA, p.(Gln61Lys)	3	L	CHSPv2	
				T3	micro-PTC	*KRAS* (exon 3), NM_033360.2: c.180_181delTCinsAA, p.(Gln61Lys)	2	L	CHSPv2	
17	f	52	+	T1	NIFTP	*NRAS* (exon 3), NM_002524.4: c.181C>A, p. (Gln61Lys)	18		CHSPv4	Archer
				T2	NIFTP	*NRAS* (exon 3), NM_002524.4: c.181C>A, p. (Gln61Lys)	16		CHSPv4	Archer
				T3	FA	*KRAS* (exon 3), NM_004985.3: c.182A>G, p. (Gln61Arg)	13		CHSPv4	Archer
18	f	44	-	T1	PTC	*BRAF* (exon 15), NM_004333.4: c.1799T>A, p.(Val600Glu)	45	R	CHSPv4 + IHC	Archer
				T2	FA	*PTEN* (exon 7), c.755A>T, p. (Asp252Val)	6	R	CHSPv4	Archer
				T3	PTC	$BRAF^{V600E}$	11	R	IHC	Archer
19	m	34	+	T1	FVPTC	*ETV6*: NM_001987.4:exon:4 ; *NTRK3*: NM_002530.3:exon:14	7	R	CHSPv4	Archer
				T2	FVPTC	*ETV6*: NM_001987.4:exon:4 ; *NTRK3*: NM_002530.3:exon:14	8	R	CHSPv4	Archer
				T3	FA	No fusion detected		R		Archer
				T4	miFA	No variant detected	12	R	CHSPv4	
20	f	46	+	T1	micro-PTC	*BRAF* (exon 15), NM_004333.4: c.1799T>A, p.(Val600Glu)	10	R	CHSPv4	Archer
				T2	micro-PTC	*BRAF* (exon 15), NM_004333.4: c.1799T>A, p.(Val600Glu)	10	R	CHSPv4	Archer
				T3	micro-PTC	*BRAF* (exon 15), NM_004333.4: c.1799T>A, p.(Val600Glu)	5	R	CHSPv4	Archer
				T4	FA		5	R	CHSPv4	
		47		T5	PTC	*BRAF* (exon 15), NM_004333.4: c.1799T>A, p.(Val600Glu)	12	L	CHSPv4†	
21	f	60	-	T1	EFVPTC	*HRAS* (exon 3), NM_001130442.1: c.181C>A, p.(Gln61Lys)	20	R	CHSPv3	
		71		T2	NIFTP	*NRAS* (exon 3), NM_002524.4: c.182A>G, p.(Gln61Arg)	10	L	CHSPv3	
				M1	FVPTC	*HRAS* (exon 3), NM_001130442.1: c.181C>A, p.(Gln61Lys)		L3§	CHSPv3	
**(Partly) shared molecular alterations (progression/subclonal diversification/other?)**										
22	f	61	-	T1	FA	*KRAS* (exon 3): NM_004985.3: c.182A>G, p.(Gln61Arg)	25	L	CHSPv6	
				T2	Oncocytic proliferation ††	*KRAS* (exon 3): NM_004985.3: c.182A>G, p.(Gln61Arg) and *TERT (promoter)*: NM_198253.2: c.-124C>T	17	L	CHSPv6	
				T3	NIFTP	*NRAS* (exon 3): NM_002524.4: c.182A>G, p.(Gln61Arg) and *SMAD4* (exon 6); : NM_005359.5: c.742_743insG, p.(Gln248Argfs*16)	2	L	CHSPv6	
23	f	38	+	T1	miFTC	*HRAS* (exon 3): NM_001130442.1: c.182A>G, p.(Gln61Arg)	10	R	CHSPv4	
				T2	FA	*HRAS* (exon 3): NM_001130442.1: c.182A>G, p.(Gln61Arg)	10	R	CHSPv4	
				T3	FA	*HRAS* (exon 3): NM_001130442.1: c.182A>G, p.(Gln61Arg)	10	R	CHSPv4	
				T4	FA	*NRAS* (exon 3): NM_002524.4: c.182A>G, p.(Gln61Arg)	19	R	CHSPv4	
				T5	FA	*NRAS* (exon 3): NM_002524.4: c.182A>G, p.(Gln61Arg)	10	R	CHSPv4	
				T6	FA		5	R		
24	f	38	-	T1a	NIFTP high cellularity	*HRAS* (exon 3), NM_001130442.1: c.182A>G, p.(Gln61Arg)	19	R	CHSPv4	Archer
				T1b	NIFTP low cellularity	*HRAS* (exon 3), NM_001130442.1: c.182A>G, p.(Gln61Arg)		R	CHSPv4	Archer
25	f	55	+	T1	NIFTP high cellularity	*HRAS* (exon 3); NM_005343.2: c.182A>G, p.(Gln61Arg)	7	R	CHSPv6	
				T2	NIFTP low cellularity	*HRAS* (exon 3); NM_005343.2: c.182A>G, p.(Gln61Arg)	7	R	CHSPv6	
				T3	micro-PTC		1.6	R		

† The molecular diagnostic results of three tumor foci (T1 and T2 of case 14; T5 of case 20) were obtained from the thyroid cytology slide.

‡ Lymph node metastasis present in cases 3 and 12.

§ Lumbar vertebra L3.

†† Inside the FA (T1) there was an oncocytic proliferation (T2) (lack of capsular/vascular invasion, but aggressive molecular profile; hence no designation as either oncocytic follicular adenoma, nor oncocytic carcinoma of the thyroid, was preferred in this specific case).

PTC, papillary thyroid carcinoma; micro-PTC, papillary thyroid microcarcinoma; FVPTC, follicular variant of papillary thyroid carcinoma; EFVPTC, encapsulated follicular variant of papillary thyroid carcinoma; NIFTP, non-invasive follicular thyroid neoplasm with papillary-like nuclear features; miFTC, minimally invasive follicular thyroid carcinoma; FA, follicular adenoma; maFA, macrofollicular adenoma; miFA, microfollicular adenoma.

+, present; -, absent; f, female; m, male; yrs, years; CLT, chronic lymphocytic thyroiditis; Diam., diameter; Loc., location; T, tumor focus; M, metastatic focus; CHSPv, Cancer Hotspot Panel version; Taq, Taqman hydrolysis assay; IHC, immunohistochemistry; Archer, Archer CTL FusionPlex; L, left lobe of thyroid gland; I, isthmus; R, right lobe of thyroid gland.

Note 1: as regards subtyping of papillary thyroid (micro)carcinoma (PTC): if not further specified, this concerns “classic papillary thyroid carcinoma” in this manuscript.
Note 2: the designation of papillary thyroid microcarcinoma (micro-PTC) was used to emphasize subcentimetric PTC foci with a diameter ≤1cm due to potential clinical implications in the current context, rather than a histologic subtype.

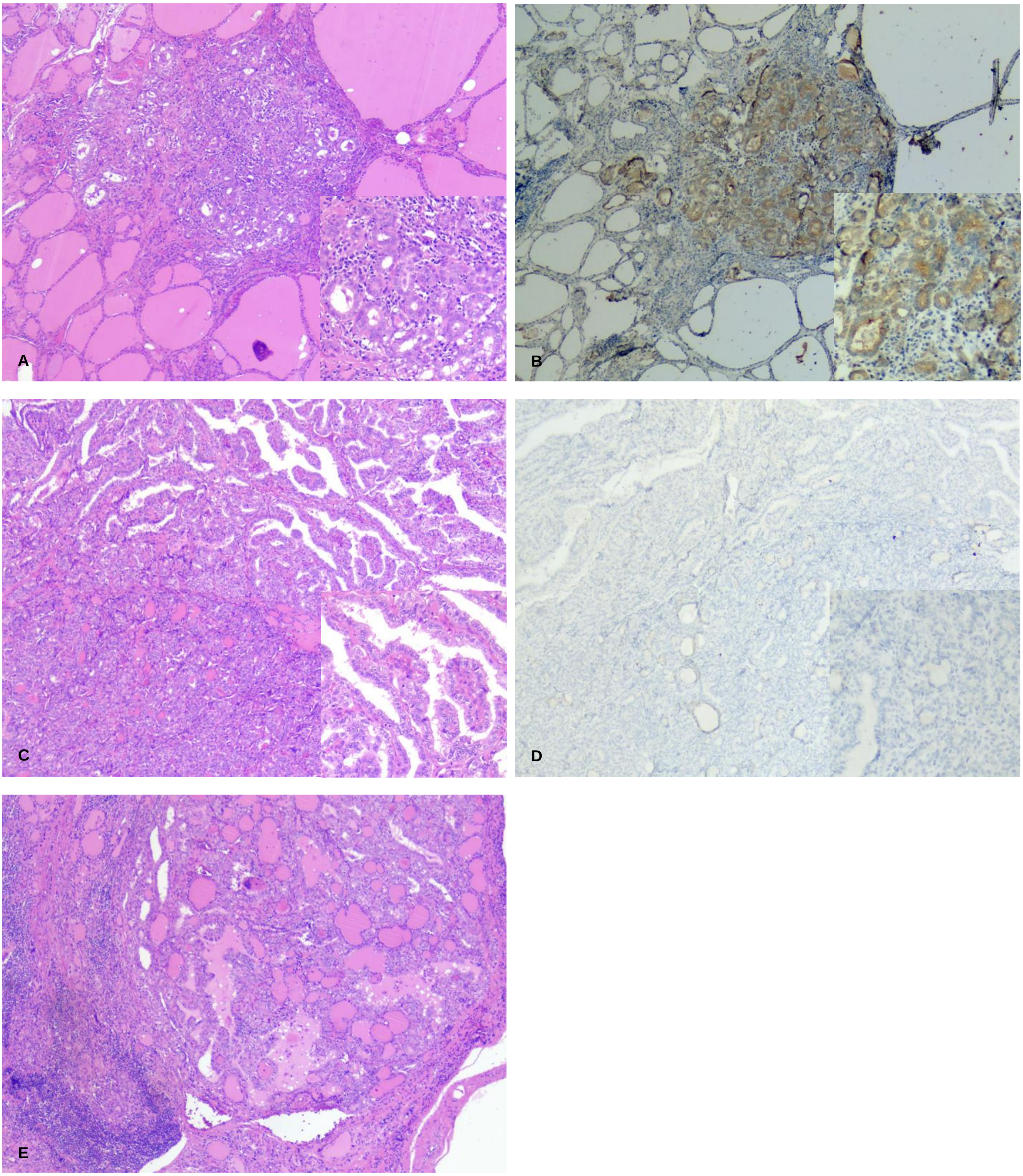

Supplementary Figure S1. Histologic photomicrographs of three separate tumor foci (**A**, **C**, **E**) of papillary thyroid microcarcinomas, with different molecular profiles each; an example of three Multi-UniFocal (MUF) micro-PTCs in patient case 2.

(**A**, H&E) Tumor focus 1 harbors a *BRAF*^{V600E} mutation, (**B**) and has positive immunohistochemical staining for BRAFVE1.

(**C**, H&E) Tumor focus 3 harbors a *BRAF*$^{non\text{-}V600E}$ gene variant, (**D**) and shows negative immunohistochemical staining for BRAFVE1.

(**E**, H&E) Tumor focus 2 harbors a *SASH1-BRAF* gene fusion. Also note the background with lymphocytic thyroiditis, as was seen widespread through the thyroid dissection. *Higher magnifications are shown as insets.*

Supplementary Table S2. Comparison of clinicopathologic characteristics in 25 patients with thyroid lesions involving Multi-UniFocality (MUF), based on coexistent chronic lymphocytic thyroiditis (CLT).

Parameters	**MUF cases with CLT** ***n* = 12 (100%)**	**MUF cases without CLT** ***n* = 13 (100%)**
Female, *n* (%)	9 (75)	8 (62)
Male, *n* (%)	3 (25)	5 (38)
Age, years (median, range)	44 (34 – 66)	51 (29 – 61)
Tumor foci per patient (median, range)	4 (2 – 6)	3 (2 – 6)
Tumor size, *n* (%)		
≤1 cm	29 (63)	22 (61)
>1 cm	17 (37)	14 (39)
Lymph node metastasis	1 (8)	1 (8)
Distant metastasis	0 (0)	1 (8)
Total thyroidectomy, *n* (%)	10 (83)	10 (77)
Unilateral lesions	4 (33)	8 (62)
Bilateral lesions	7 (58)	5 (38)
Histology, *n* (%)		
PTC (including micro-PTC)	7 (58)	8 (62)
micro-PTC	7 (58)	6 (46)
FVPTC	3 (25)	3 (23)
EFVPTC	0	4 (31)
NIFTP	2 (17)	6 (46)
FA	8 (67)	4 (31)
Oncocytic proliferation focus	0	1 (8)
miFTC	2 (17)	0
Gene variants, *n* (%)		
$BRAF^{V600E}$	5 (42)	5 (38)
$BRAF^{non\text{-}V600E}$	1 (8)	0
MAP2K1	1 (8)	0
(H-/K-/N-)RAS	6 (50)	12 (92)
– HRAS	3 (25)	3 (23)
– KRAS	1 (8)	5 (38)
– NRAS	5 (42)	8 (62)
PTEN	0	1 (8)
SMAD4	0	1 (8)
TP53	0	1 (8)
TERTp	0	1 (8)
Gene fusions, *n* (%)		
BRAF	1 (8)	0
RET	1 (8)	0
NTRK	1 (8)	0
PPARG	1 (8)	2 (15)
MET	1 (8)	0

Of note, in this table the frequencies of the variables are shown as per patient case. (Although multiple tumor foci with the same histology or gene variant may be present in one patient case, the frequency will not be noted per tumor focus, but as ‘1’ per patient case.)

PTC, papillary thyroid carcinoma; micro-PTC, papillary thyroid microcarcinoma; FVPTC, follicular variant of papillary thyroid carcinoma; EFVPTC, encapsulated follicular variant of papillary thyroid carcinoma; NIFTP, non-invasive follicular thyroid neoplasm with papillary-like nuclear features; miFTC, minimally invasive follicular thyroid carcinoma; FA, follicular adenoma.

Note 1: as regards subtyping of papillary thyroid (micro)carcinoma (PTC): if not further specified, this concerns “classic papillary thyroid carcinoma” in this manuscript.
Note 2: the designation of papillary thyroid microcarcinoma (micro-PTC) was used to emphasize subcentimetric PTC foci with a diameter ≤1cm due to potential clinical implications in the current context, rather than a histologic subtype.

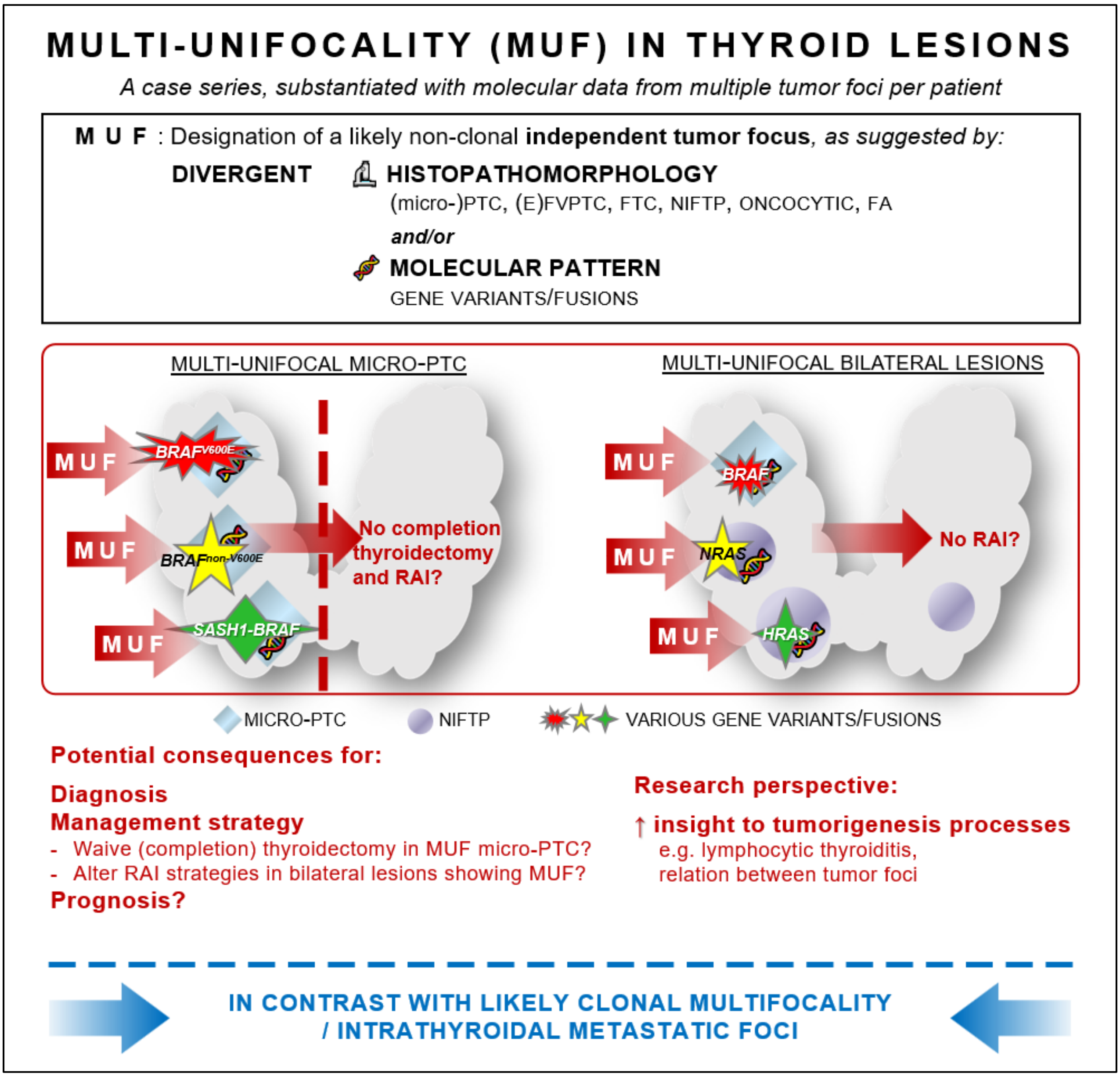

Supplementary Figure S2. The recognition of 'UniFocality within Multifocal thyroid lesions' (Multi-UniFocality, MUF) may justify the independent clinical consideration per individual tumor focus; as a separate lesion. Accordingly, the recognition of MUF may potentially improve diagnosis and alter further clinical management in select cases, as illustrated by the present case series. *Graphical impressions based on cases 2 and 7 (shown on the left and right, respectively).*

Additional discussion on laterality

Another interesting discussion point might be whether bilaterally or unilaterally arisen tumors can be correlated with (in)dependent clonal origin or some prognostic value. In their retrospective study on 635 PTC patients, Feng et al. reported on multifocal (n=157, 24.7%) and bilateral (n=99, 15.6%) PTC; while multifocality was associated with an increased risk of recurrence, bilaterality was not (as set out against unilateral multifocality). Moreover, this risk of recurrence increased the more tumor foci were present, for multifocal PTC overall, but also for the multifocal non-microcarcinoma cases (with maximum diameter PTC focus >1cm); whereas this was not the case with the multifocal papillary thyroid microcarcinoma patients. (8) Zhang et al. associated unilateral multifocal PTC with more lymph node metastasis, advanced TNM stage and recurrent/persistent disease than unilateral unifocal PTC in their meta-analysis (5). Wang et al. found in their retrospective study with more than 2000 consecutive PTC patients, that bilateral PTC (n=425, 19.2%) was more aggressive than unilateral-multifocal PTC (n=210, 9.5%) (59). Also Yan et al. found in their retrospective study on papillary thyroid microcarcinoma (n=3005) that bilateral multifocal (n=573), rather than unilateral multifocal papillary thyroid microcarcinoma (n=272), was more aggressive (extrathyroidal extensions, lymph node metastasis) compared to unifocality, however no independent prognosticator (38). Again, since multifocality is not further specified, the results could also possibly be a reflection of intrathyroidal metastatic multifocality and not necessarily representative of MUF, in hypothesis.

In terms of clonality, Wang et al. reported that the majority in their study of 25 synchronous/metachronous bilateral multifocal PTC arose from a single clone, underscoring the important role of intrathyroidal metastasis in the development of bilateral and recurrent disease (53). Bansal et al. found in their study that tumors with different mutations were associated with occurrence in opposite lobes, as opposed to tumors with identical mutations (14). Shattuck et al. reported 3 of 7 bilateral multifocal PTC as being from independent clonal origins (48). Furthermore, Lu et al. reported 6 out of 8 multifocal PTC cases as bilateral, of which 3 cases of independent clonal origin, 2 cases of common clonal origin, 1 case with co-occurring independent and common clonal origins. Lu et al. further reported independent clonality in 2 unilateral cases. (1) Also in the present case series, there were both cases with unilateral and bilateral Multi-UniFocality.

Still, as exemplified by the studies mentioned above and the present study, the assignment of clonal origin status to bilateral or unilateral multifocal cases may be rather ambiguous due to co-occurring independent and common clonal origin, but also incomplete molecular profiles of every single tumor focus. Another point of consideration is the potential role of time or tumor progression/recurrence in the likelihood for bilateral disease.
