# Supplementary material for: Multi‐UniFocality (MUF), in contrast to multifocality, in thyroid lesions: Relation to lymphocytic thyroiditis
Source: Pathol Int. 2024 Apr 1;74(5):274–84. doi: 10.1111/pin.13421 (PMC11551814; doi:10.1111/pin.13421)
Supplement: Supplementary file 2 — Supporting information. [file PIN-74-274-s002.docx]

**List of Supplementary Material**

**Supplementary Table S1.** Case series of patients with thyroid lesions involving Multi-UniFocality (MUF), full data.

**Supplementary Figure S1.** Histologic photomicrographs of three separate tumor foci (**A**, **C**, **E**) of papillary thyroid microcarcinomas, with different molecular profiles each; an example of three Multi-UniFocal (MUF) micro-PTCs (patient case 2).

(**A**, H&E) Tumor focus 1 harbors a *BRAF^V600E^* mutation, (**B**) and has positive immunohistochemical staining for BRAFVE1 . (**C**, H&E) Tumor focus 3 harbors a *BRAF^non-V600E^* gene variant, (**D**) and shows negative immunohistochemical staining for BRAFVE1. (**E**, H&E) Tumor focus 2 harbors a *SASH1-BRAF* gene fusion. Also note the background with lymphocytic thyroiditis, as was seen widespread through the thyroid dissection. *Higher magnifications are shown as insets.*

**Supplem****entary Table S2.** Comparison of clinicopathologic characteristics in 25 patients with thyroid lesions involving Multi-UniFocality (MUF), based on coexistent chronic lymphocytic thyroiditis (CLT).

**Supplementary Figure S2.** The recognition of ‘unifocality within multifocal thyroid lesions’ (Multi-UniFocality, MUF) may justify the independent clinical consideration per individual tumor focus; as a separate lesion. Accordingly, the recognition of MUF may potentially improve diagnosis and alter further clinical management in select cases, as illustrated by the present case series. *Graphical impressions based on cases 2 and 7, (shown on the left and right respectively.)*

**Supplementary material.** Additional discussion on laterality.
